# Copy number variations contribute to malignant tumor development in children with serious birth defects

**DOI:** 10.1002/1878-0261.13718

**Published:** 2024-08-14

**Authors:** Yichuan Liu, Joseph Glessner, Hui‐Qi Qu, Xiao Chang, Haijun Qiu, Tiancheng Wang, Frank D. Mentch, Hakon Hakonarson

**Affiliations:** ^1^ Center for Applied Genomics (CAG) Children's Hospital of Philadelphia PA USA; ^2^ Department of Pediatrics, The Perelman School of Medicine University of Pennsylvania Philadelphia PA USA; ^3^ Division of Human Genetics Children's Hospital of Philadelphia PA USA; ^4^ Division of Pulmonary Medicine Children's Hospital of Philadelphia PA USA; ^5^ Faculty of Medicine University of Iceland Reykjavík Iceland

**Keywords:** birth defects, copy number variations, pediatric cancers, whole‐genome sequencing

## Abstract

There are two key signatures of pediatric cancers: (a) higher prevalence of germline alterations and (b) heterogeneity in alteration types. Recent population‐based assessments have demonstrated that children with birth defects (BDs) are more likely to develop cancer even without chromosomal anomalies; therefore, explorations of genetic alterations in children with BDs and cancers could provide new insights into the underlying mechanisms for pediatric tumor development. We performed whole‐genome sequencing (WGS) on blood‐derived DNA for 1556 individuals without chromosomal anomalies, including 454 BD probands with at least one type of malignant tumor, 757 cancer‐free children with BDs, and 345 healthy individuals, focusing on copy number variation (CNV) analysis. Roughly half of the children with BD‐cancer have CNVs that are not identified in BD‐only/healthy individuals, and CNVs are not evenly distributed among these patients. Strong heterogeneity was observed, with a limited number of cancer predisposition genes containing CNVs in more than three patients. Moreover, functional enrichments of genes with CNVs showed that dozens of patients have variations related to the same biological pathways, such as deletions of genes with neurological functions and duplications of immune response genes. Phenotype clustering uncovered recurrences of patients with sarcoma: A notable enrichment was observed involving non‐coding RNA regulators, showing strong signals related to growth and cancer regulations in functional analysis. In conclusion, we conducted one of the first genomic studies exploring the impact of CNVs on cancer development in children with BDs, unveiling new insights into the underlying biological processes.

## Graphical abstract

Children with birth defects are more likely to develop cancer. We observed strong heterogeneity and uneven distributions of copy number variations (CNVs) in 454 such children, with limited occurrence of cancer predisposition genes. However, consistency of the functional impact of CNVs on pathways, and enrichment of non‐coding RNA, was observed, showing strong signals related to growth and cancer regulation.

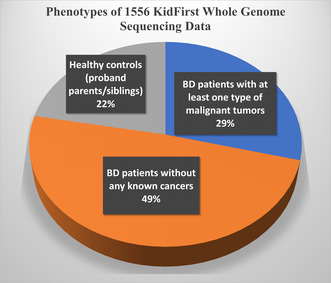

**Abbreviations**
BDs: birth defects
CAG: Center for Applied Genomics
CHOP: Children's Hospital of Philadelphia
CNV: copy number variation
DRAGEN: Dynamic Read Analysis for GENomics
EMRs: electronic medical records
ICD code: International Classification of Diseases code
LncRNAs: long non‐coding RNAs
VCF: variant call format
WebGestalt: WEB‐based Gene SeT AnaLysis Toolkit
WGS: whole‐genome sequencing

## 1 Introduction

Pediatric cancers have fewer somatic mutations, but a higher prevalence of germline alterations compared to cancers in adults. Strong heterogeneity of the mutations plus limited genome profiling at diagnosis has halted progression of our understanding of the underlying mechanisms of malignant tumor development and progression in pediatric cancer patients [1]. It is well known that children with birth defects (BD) are more likely to develop cancers in their later life, with large population‐based studies showing that even without chromosomal anomaly, the risks are still 2.5‐fold higher in children with BD compared to children without BD. As such, the risk of cancers increased significantly in children with BD regardless of any underlying chromosomal anomalies [2, 3]. BDs affect 1 in 33 newborns in the United States each year based on the most recent data from Centers for Disease Control and Prevention (CDC; https://www.cdc.gov/birth‐defects/data‐research/facts‐stats/index.html).

According to data from United Nations, Department of Economic and Social Affairs, Population Division, there are more than 3.7 million newborns alive in 2022 in the United States, which suggests more than 110 000 children are born with significant BD. Considering the significant effect size, the cancer risk in children with BD without chromosomal anomaly cannot be neglected.

Hypotheses have been made for the high prevalence of pediatric tumors in BD, in particular the “two hit hypothesis” with BD as the first hit [4, 5]. At the genetic level, previous studies suggest that pediatric tumors may be associated with developmental disruptions rather than carcinogenic exposures [6, 7]. Although multiple large cohort studies have confirmed the prevalence of malignant tumors in children with BD across different populations [4, 8, 9], few studies have evaluated the shared molecular pathways between pediatric tumors and BD. Specifically, there is a lack of research investigating the associations between genomic structural variations and the phenotypes. We hypothesize that pediatric cancers may result from aberrant genetic control of fetal development and thus share a common etiology with BDs. Copy number variations (CNVs) are types of structural variation, including duplication or deletion events that affect a variable number of base pairs. Previous studies have shown that CNVs are critical contributions in adult cancers and often serve as biomarkers in the diagnosis, monitoring of disease progression, and selection of targeted medicines in pediatric cancers [4, 5, 6]. To assess the potential impact of CNVs and their roles in pediatric cancer development in children with BDs, we performed whole‐genome sequencing (WGS) in a large cohorts of BD patients with or without cancer, as a part of Gabriella Miller Kids First project (https://commonfund.nih.gov/kidsfirst).

## 2 Materials and methods

### 2.1 Patient recruitment

The patients with birth defects (BDs), with or without co‐occurrence of pediatric onset cancers, were recruited by the Center for Applied Genomics (CAG), the Children's Hospital of Philadelphia (CHOP). The BD and cancer diagnosis used the International Classification of Diseases (ICD) codes ICD‐9/ICD‐10. All the CAG patients were recorded in the electronic medical records (EMRs) of CHOP established in 2003. CAG at CHOP maintains a de‐identified extract of clinical data from the CHOP EMR and EHR databases of consented patients. This database contains longitudinal information about visits, diagnoses, medical history, prescriptions, procedures, and lab tests with all information coded and de‐identified.

**Fig. 1:**
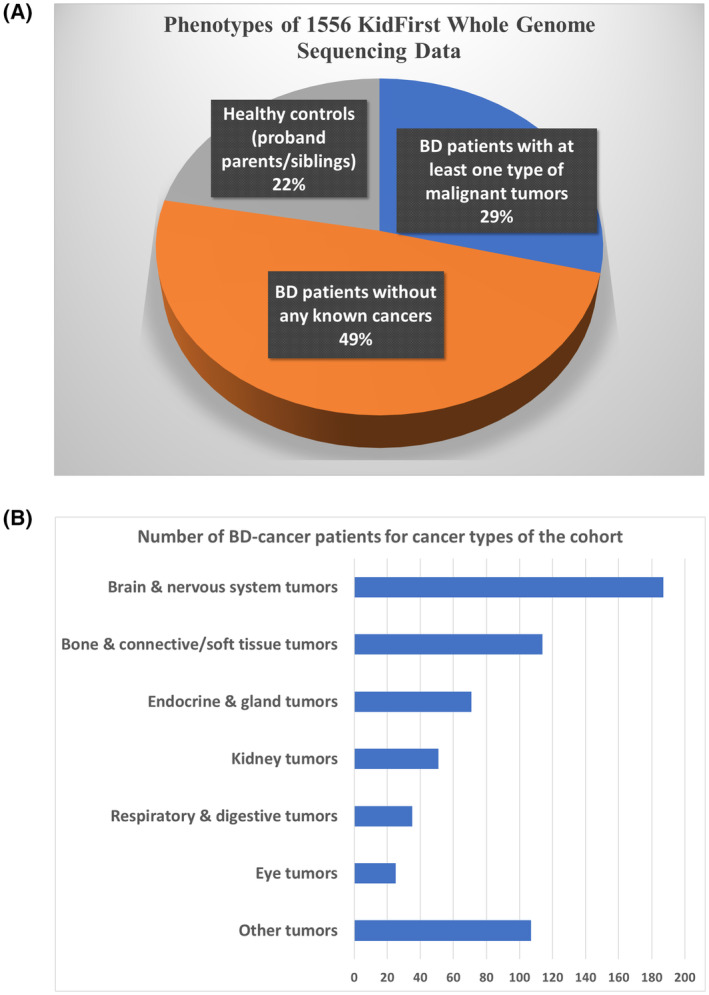
Summaries of phenotypes of KidFirst cohorts. (A) Pie‐chart of number children with birth defects (BDs) diagnosed with at least one type of malignant tumors; (B) number of children with BD‐cancer in different categories of pediatric cancers.

Altogether, 1211 probands with non‐chromosomal anomalies and at least one major BD were studied (Table S1a), including 454 BD patients with at least one type of malignant tumors (BD‐cancer, Table S1b) and 757 BD patients without any known cancers (BD‐only). A patient may have multiple BDs. The most common BD/cancer is 40 patients with neurofibromatosis, type 1 with malignant neoplasm of other and unspecified parts of nervous system (Q85.01/192), and 33 patients with other specified congenital anomalies of brain with malignant neoplasm of brain, unspecified (742.4/191.9). Probands diagnosed with any types of hematologic malignancies were excluded. In addition, 345 healthy controls without BD or cancer who were parents/siblings of the probands were also investigated in comparison (Fig. 1). All the patients were recruited during regular hospital visits at multiple clinical settings, including emergency room, ambulatory settings or surgical settings, through the general pediatric clinics or CHOP's pediatric specialty practices. The patients were in the age range of 0–21 years and receiving health care at CHOP. Parental consent was obtained for individuals under 18 years of age, and assent was also obtained for subjects aged 7–17 years. The consent allows samples to be obtained and analyzed using the genomic technologies in this study, to address the proposed research questions.

We confirm that all research and involved methods were carried out in accordance with relevant guidelines and regulations and all experimental protocols involved were approved by the Children's Hospital of Philadelphia (CHOP) Institutional Review Board (IRB 16‐013278). Written informed consent was obtained from all subjects or, if subjects are under 18 years, from a parent and/or legal guardian with assent from the child if 7 years or older. The study methodologies conformed to the standards set by the Declaration of Helsinki. The samples were collected from 2016 to 2021.

### 2.2 Sample processing and variant detection by WGS

Whole‐genome sequencing (WGS) was done at 30× coverage for the 1566 individuals as a part of the Gabriella Miller Kids First project, by the genomics platform of the Broad Institute. The variant call format (VCF) files of WGS were generated using the Illumina DRAGEN (Dynamic Read Analysis for GENomics) Bio‐IT Platform (Illumina, San Diego, CA, USA), aligned to the GRCh38/hg38 human genome assembly. The annotations for the variants were generated using the ANNOVAR software developed by our group [7], and the variants were further classified into variants in coding regions, introns, 5′ or 3′ untranslated regions (UTR), and non‐coding RNA regions. The recruitment processes and sample processing have also been described in our earlier paper [10].

### 2.3 Detections of CNVs

The detections of CNVs were achieved by four independent CNV callers, including MANTA [8], CNVnator [9], DELLY [11], and LUMPY [12]. Then, the detected CNVs were combined based on following criteria: (a) the maximum distance between breakpoints is not exceeding 1000 bp; (b) at least three CNV callers support the detected CNV; and (c) the minimum size of the CNVs is over 10 000 bp. The combined results of CNVs were further filtered based on multiple thresholds: (a) MANTA quality score is at least 100; (b) DELLY quality score is at least 1000; and (c) alt allele count is < 5%. The remaining CNVs were pursued for downstream analysis.

### 2.4 Functional impacts of CNVs

For protein‐coding genes and non‐coding RNAs (ncRNAs) identified based on the GRCh38/hg38 GENCODE human genome reference, they were categorized into full gene deletions, full gene duplications, partial gene deletions, and partial gene duplications based on the size of the CNV, respectively. More specifically, if a protein‐coding/non‐coding gene is located inside a CNV deletion at a genomic locus, it is considered as full gene deletion; if a protein‐coding/non‐coding gene overlapped with a CNV deletion but not entirely covered, it is considered as partial gene deletion, same for duplications. To explore the potential mechanisms related to cancer development and progressions in children with BD, we identified the coding/non‐coding genes impacted by CNV in BD‐cancer patients that were absent in BD‐only patients and healthy family controls. Targets/regulators of ncRNAs, especially long non‐coding RNAs (lncRNAs), were calculated based on LncTarD version 2.0 [13], a manually curated database that provides key lncRNA–target regulations and lncRNA‐mediated regulatory mechanisms in human diseases. Functional enrichment analysis of selected genes was performed using the WebGestalt (WEB‐based Gene SeT AnaLysis Toolkit) [14].

## 3 Results

### 3.1 Selected CNVs and corresponding genes among BD‐cancer patients

**Fig. 2:**
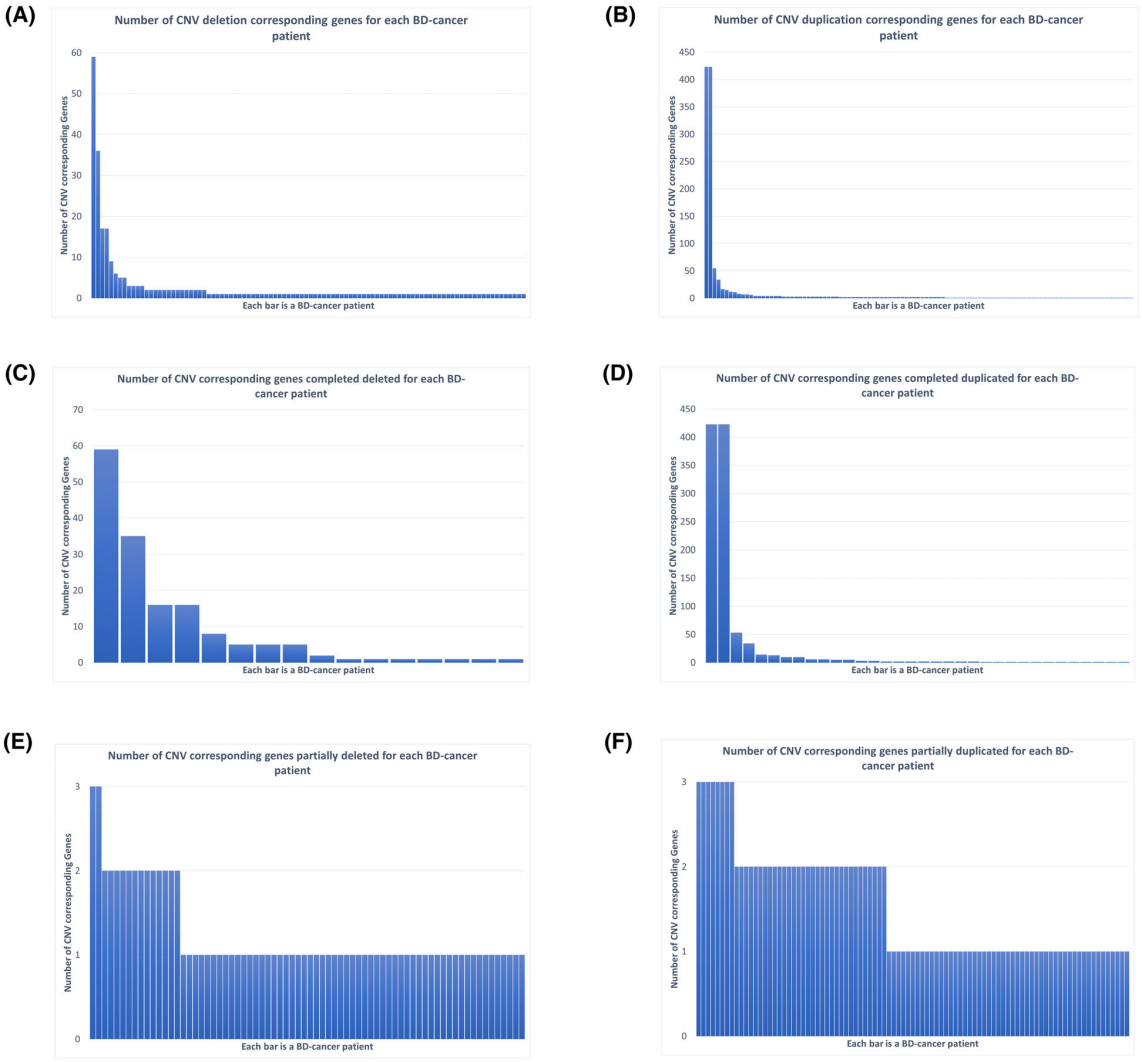
Number of copy number variation (CNV) corresponding genes in each patient with BD‐cancer. Each bar is a BD‐cancer patient, and *y*‐axis is count of genes impacted by CNVs. (A) Genes contain CNV deletions; (B) genes contain CNV duplications; (C) genes were completely deleted by CNVs; (D) genes were completely duplicated by CNVs; (E) genes were partially deleted by CNVs; and (F) genes were partially duplicated by CNVs.

After merging CNV outputs from multiple tools and filtering them based on the criteria described in Section 2, 21 560 CNVs remained, including both deletions and duplications (Table S2). Among deletion CNVs, 239 corresponding protein‐coding genes were identified that associated with tumor development/progressions in 98 BD‐cancer children, and 745 duplication CNV corresponding protein‐coding genes were found in 105 BD‐cancer patients. Overall, 181 BD‐cancer patients (39.8%) contain at least one CNV associated with cancers that is absent in cancer free BD patients and not represented in healthy family controls. Strong heterogeneity of CNV corresponding protein‐coding genes was observed in both deletions and duplications, including three CNV deletion corresponding genes (*KCND2*, *SDK1*, *SP4*), each present in three or more BD‐cancer patients, and 11 CNV duplication corresponding genes (*SNCAIP*, *RAPGEF6*, *LARP1*, *FAXDC2*, *CNOT8*, *GEMIN5*, *MRPL22*, *KIF4B*, *SGCD*, *NEURL1B*), present in three or more BD‐cancer patients. Also, highly asymmetric distributions of CNV corresponding genes were observed for BD‐cancer patients (Fig. 2A,B), with some BD‐cancer patients contain a several cancers associated CNVs and some BD‐cancer patients only contain a few or a single cancer associated CNVs.

### 3.2 Functional analysis of full and partially deleted/duplicated genes in BD‐cancer patients

**Fig. 3:**
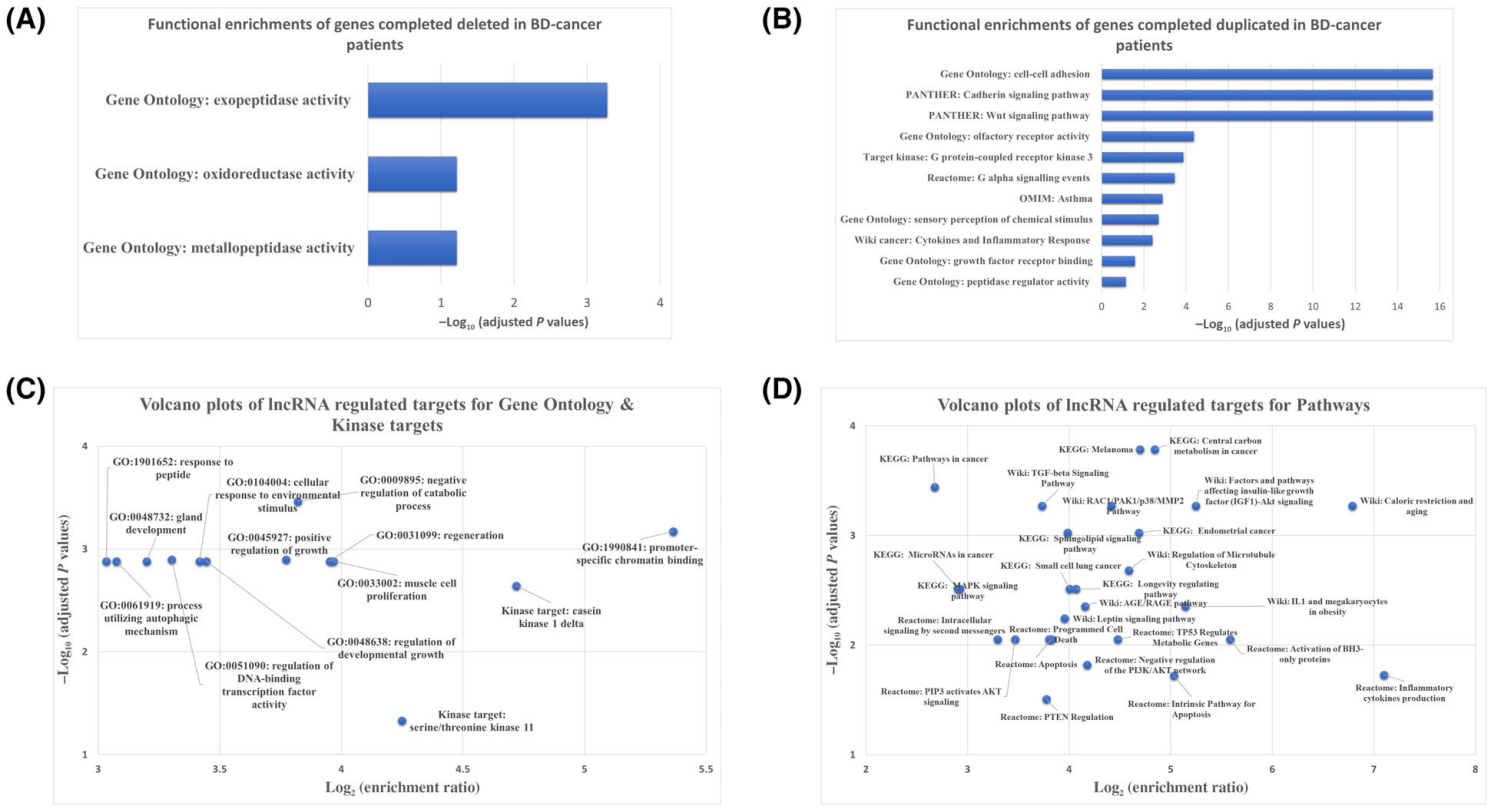
Functional enrichment analysis. (A) Bar chart for genes that were completely deleted by copy number variations (CNVs) in scale of −log_10_ (adjusted *P* value); (B) bar chart for genes were completely duplicated by CNVs; (C) volcano plots of functional enrichments of CNV impacted long non‐coding RNA (lncRNA) regulated genes in Gene Ontology (GO); and (D) volcano plots of functional enrichments of CNV impacted lncRNA regulated genes in biological pathways.

The CNV corresponding protein‐coding genes in BD‐cancer patients were further stratified into “fully” and “partially” based on their genomic locus overlap with selected CNV, as described in Section 2. The patterns for asymmetric distributions of CNV corresponding genes were consistent for genes which are fully deleted or duplicated (Fig. 2C,D). In contrast, genes that are partially deleted/duplicated are more uniform among patients (Fig. 2E,F). Collectively, 143 protein‐coding genes were fully deleted in 16 BD‐cancer patients compared to BD‐only patients, and 607 protein‐coding genes were fully duplicated in 34 BD‐cancer patients (Table S3). For genes fully deleted in BD‐cancer patients but not in BD‐only and healthy patients, functional enrichments include exopeptidase activity (FDR = 0.00053), metallopeptidase activity (FDR = 0.061), and oxidoreductase activity (FDR = 0.061). For genes fully duplicated in BD‐cancer patients but not in BD‐only and healthy patients, functional enrichments identified include cell–cell adhesion (FDR = 2.2E‐16), sensory perception of chemical stimulus (FDR = 0.0021), olfactory receptor activity (FDR = 4.3E‐5), growth factor receptor binding (FDR = 0.027), peptidase regulator activity (FDR = 0.072), Wnt signaling pathway (FDR = 2.2e‐16), cadherin signaling pathway (FDR = 2.2e‐16), and cytokines and inflammatory response (FDR = 0.0039) (Fig. 3A,B).

Collectively, 83 protein‐coding genes were partially deleted in 72 BD‐cancer patients compared to BD‐only patients, and 136 genes were partially duplicated in 91 BD‐cancer patients (Table S1). Compared to genes fully deleted/duplicated, genes partially deleted/duplicated show weaker enrichments: neurofibromatosis (FDR = 0.04) for deleted genes and synaptic membrane (FDR = 0.042) for duplicated genes. There are 13 genes (*RYR2*, *IQCM*, *CTNND2*, *SDK1*, *MSR1*, *LINGO2*, *MALRD1*, *VTI1A*, *UNC13C*, *CACNA1H*, *AC104151.1*, *ZNF100*, *CBFA2T2*) that contain partial CNV deletions in both BD‐cancer and BD‐only patients, but the deleted parts are different, and they show good trend of enrichment in synapse organization among the BD‐cancer cases (FDR = 0.071). Similarly, seven genes (*SGCZ*, *KANK1*, *KDM4C*, *KCNMA1*, *ZNF263*, *CDH4*, *NCAM2*) contain partial CNV duplications in both BD‐cancer and BD‐only patients, but the deleted loci are different. A notable finding is that genes with CNV deletions and/or duplications are enriched as target genes of microRNAs (miRs), including miR‐219, miR‐511, miR‐181, miR‐183, and miR‐330, which are known as highly functional miRs in tumor development processes.

### 3.3 Long non‐coding RNAs contain CNVs associated with BD‐cancer

Based on same criteria for protein‐coding genes, 186 lncRNAs were fully deleted in 35 BD‐cancer patients compared to BD‐only and healthy individuals, and 614 lncRNAs were fully duplicated in 59 BD‐cancer patients. In addition, 72 lncRNAs were partially deleted in 58 BD‐cancer patients and 51 lncRNAs were partially duplicated in 41 BD‐cancer patients (Table S4). For partially deleted CNVs observed in both BD‐cancer and BD‐only patients, the CNVs resided on different genomic loci, including six lncRNA deletions (*LINC00276*, *ENSG00000228999*, *ENSG00000246090*, *ENSG00000226197*, *MIR4300HG*, *ENSG00000259995*) and six lncRNA duplications (*LINC01320*, *ENSG00000231918*, *LINC02540*, *ENSG00000224972*, *ENSG00000226197*, *LINC01235*). Functional analysis of the CNV impact on the lncRNAs was done by mapping lncRNAs to their regulated targets based on experimental database information, LncTarD, as described in method sections. Collectively, 35 lncRNAs present in the database mapped to 31 protein‐coding genes and four non‐coding genes (Table 1). The gene targets involved are associated with different cancers, and functional analysis shows enrichments in cancer related terms such as positive regulation of growth (FDR = 0.0013), pathways in cancer (FDR = 0.0036), and apoptosis (FDR = 0.0089) (Fig. 3C,D).

**Table 1:** BD‐cancer lncRNAs impacted by CNVs that identified with known regulated targets.

LncRNA ID	CNVs	Regulated targets	Targets associated diseases
LINC00115	Fully deletion	ZNF596	Malignant glioma
LINC01128	Fully deletion	SFN	Cervical cancer
LINC‐PINT	Fully deletion	PTCH1	Acute lymphocytic leukemia, gastric cancer, esophageal cancer, non‐small cell lung cancer, osteosarcoma, laryngeal carcinoma
LINC01189	Fully deletion	miR‐155‐5p	Hepatitis C
LINC01093	Fully duplication	GLI1	Hepatocellular carcinoma
LUCAT1	Fully duplication	NRF2	Hepatoblastoma, osteosarcoma, clear cell renal cell carcinoma, non‐small cell lung cancer, esophagus squamous cell carcinoma, thoracoabdominal aorta aneurysm, breast cancer, renal fibrosis, chronic heart failure, chronic obstructive pulmonary disease, pancreatic cancer, hepatocellular carcinoma, pancreatic ductal adenocarcinoma, cervical cancer, colorectal cancer, triple‐negative breast cancer, ovarian cancer, gastric cancer, papillary thyroid carcinoma
NR2F1‐AS1	Fully duplication	SIK1	Osteosarcoma, endometrial cancer, gastric cancer, breast cancer lung metastatic dormancy, thyroid cancer, cervical squamous cell carcinoma
MCTP1‐AS1	Fully duplication	miR‐650	Endometrial cancer
LINC01554	Fully duplication	PKM	Esophagus squamous cell carcinoma, hepatocellular carcinoma
RGMB‐AS1	Fully duplication	NLRP3	Lung adenocarcinoma, non‐small cell lung cancer, gastric cancer, laryngeal squamous cell carcinoma
LINC00491	Fully duplication	ROCK1	Hepatocellular carcinoma
EPB41L4A‐AS1	Fully duplication	MYD88	Cancer, type 2 diabetes mellitus
EPB41L4A‐DT	Fully duplication	FOXL1	Hepatocellular carcinoma
LINC02200	Fully duplication	NFKB1	Sinonasal squamous cell carcinoma
LINC01170	Fully duplication	AKT1	Endometrial cancer
MIR3936HG	Fully duplication	STMN1	Nasopharynx carcinoma, gastric cancer
WSPAR	Fully duplication	miR‐200c	Asthma, colorectal cancer, malignant glioma, hepatocellular carcinoma, non‐small cell lung cancer, kidney disease
EPIST	Fully duplication	CYC1	Osteosarcoma, hepatocellular carcinoma, oral squamous cell carcinoma
SMAD5‐AS1	Fully duplication	SMAD5	Diffuse large B‐cell lymphoma, nasopharynx carcinoma
SNHG4	Fully duplication	MET	Osteosarcoma, neuroblastoma, renal cell carcinoma, acute myeloid leukemia, non‐small cell lung cancer, neonatal pneumonia, prostate cancer, cervical cancer, lung cancer
MALINC1	Fully duplication	PURA	Cancer
CARMN	Fully duplication	miR‐21	Urinary bladder cancer, laryngeal squamous cell carcinoma
CLMAT3	Fully duplication	CDH1	Colorectal cancer
SAP30L‐AS1	Fully duplication	SAP30L	Prostate cancer
FAM87A	Fully duplication	PPM1H	Malignant glioma
B4GALT1‐AS1	Fully duplication	YY1AP1	Sepsis‐induced acute kidney injury
LINC00842	Fully duplication	PPARGC1A	Pancreatic ductal adenocarcinoma
FAM99A	Fully duplication	YAP1	Pre‐eclampsia
NRIR	Partially deletion	FUBP3	Colorectal cancer
PURPL	Partially deletion	TP53	Colorectal cancer, liver cancer
TRG‐AS1	Partially deletion	SUZ12	Glioblastoma
LINC01410	Partially deletion	CHD7	Thyroid cancer, gallbladder cancer, endometrial cancer
TCL6	Partially deletion	PTEN	Pre‐eclampsia, threatened abortion, retinoblastoma
LINC01579	Partially deletion	EIF4G2	Glioblastoma
LINC00922	Partially deletion	LZTS1	Lung cancer, colorectal cancer

### 3.4 Clustering of BD‐cancer with CNVs based on enriched pathways and phenotype

**Fig. 4:**
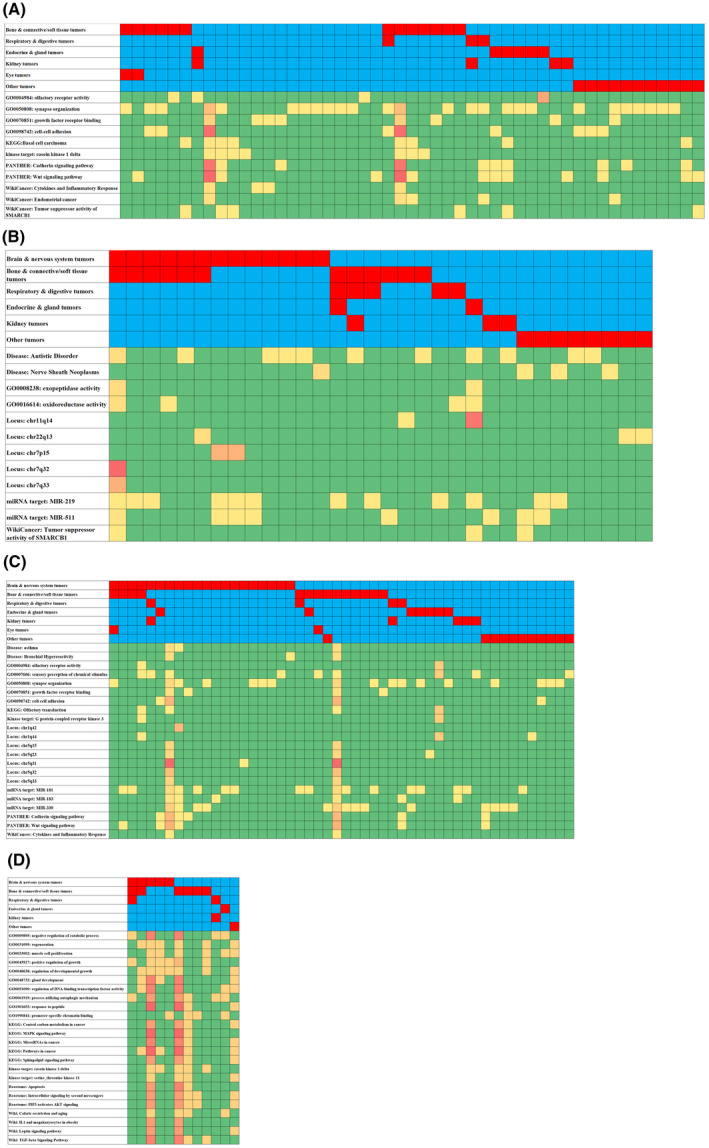
Clustering of birth defect (BD)‐cancer patients based on different diagnosis and copy number variation (CNV) impacted genes/long non‐coding RNA (lncRNA) functional enriched terms. (A) For all CNV detected; (B) CNV deletions; (C) CNV duplications; and (D) lncRNA targeted genes. The color is the heat of number of patients in corresponding diagnosis/functional categories; red color indicates higher occurrence, and blue means lower/no occurrences.

Due to heterogeneity and asymmetric distribution of CNVs in BD‐cancer patients, we further clustered the phenotype of BD‐cancer patients based on two dimensions: functional enrichment hits of CNV corresponding protein‐coding/non‐coding genes for deletions and duplications, respectively, and cancer phenotypes identified by ICD code, as described in the Method section. As shown in Fig. 4, 32 BD‐cancer patients containing at least one CNV deleted gene mapped to at least one significant functional pathway, as well as 50 BD‐cancer patients for CNV duplications. When assessing functional biological consequences involving the genes captured with CNVs, we identified enrichment in certain rare recurrent CNVs involving cancer promoting pathways. Interestingly, the CNVs uncovered in the BD‐cancer patients were different from those in the BD cases without cancer, suggesting they may have different biological consequences that are cancer promoting.

## 4 Discussion

The exploration of underlying mechanisms driving the development of pediatric cancers in patients with BDs remains an area of limited investigation. This scarcity of research arises from the fact that diagnosis is typically established only after the emergence of malignant tumors. Indeed, the majority of studies have focused on genetically profiling pediatric cancers solely at the time of diagnosis and little attention has been paid to underlying BD. This approach has led to an incomplete understanding of disease progression, resistance to therapy, and the process of metastasis. As a consequence, the study of neoplasms and malignant cancer within children with BD, especially those lacking known chromosome anomalies, is even more scarce. Conversely, recent comprehensive population studies encompassing over 10 million live births have revealed that even non‐chromosomal BDs are linked to a roughly 2.5‐fold increase in pediatric cancer risk. Given the substantial impact of BD (as indicated by approximately 3% occurrence rate in the United States), the potential cancer risk among children with BD devoid of chromosomal anomalies cannot be ignored. While CNVs have been established as influential factors in adult cancers and hold promise as biomarkers for diagnosis, progression, and targeted therapies in pediatric cancers, their functions and impacts within pediatric cancers—particularly in children with BDs—have remained inadequately addressed. This study seeks to provide an initial glimpse into the impacts of CNVs on a genomic scale, drawing from one of the most extensive resources of pediatric BD and oncology data.

Methodologically, the study identified CNVs using whole‐genome sequencing data from 1211 probands exhibiting non‐chromosomal anomalies. This dataset comprised 454 BD patients with at least one form of malignant tumor (referred to as BD‐cancer), along with 757 BD patients who showed no evidence of cancer (referred to as BD‐only). An additional control group consisted of 345 healthy individuals who were parents or siblings of the probands. To mitigate false‐positive CNVs resulting from their benign prevalence in the human genome, four widely recognized CNV calling algorithms were employed, and their results were harmonized within specific thresholds. Instead of focusing solely on the CNV loci, the study emphasized the effects of CNVs on the respective genes—whether these genes were fully or partially deleted or duplicated. Following this methodology, 181 out of 454 (39.8%) BD‐cancer patients were identified as harboring at least one CNV after filtration. Notably, there was an uneven distribution of CNVs among patients.

It is important to note that for the CNVs in BD‐cancer patients, the blood samples were obtained at the time of hospital visits rather than at birth. Since the BD‐cancer patients were diagnosed with at least one type of malignant tumor, it is possible that the visits were due to the symptoms and/or comorbidities of the cancers. As a result, the chance of impacting the low number of CNVs was minimal. In this study, we excluded all patients with leukemia. Technically, we applied a powerful integration algorithm to eliminate false‐positive CNVs, for example, by cell‐free DNA in the blood released from solid tumors. Meanwhile, the results further illuminated a notable heterogeneity in the contributions of CNVs to BD‐cancer development. This heterogeneity was particularly evident in the observation that while some children exhibited a significant count of CNVs, approximately 60% of patients showed no valid CNVs. Furthermore, the recurrence of CNVs impacting the same gene across distinct BD‐cancer patients was relatively infrequent. For CNV deletions, only three genes (*KCND2*, *SDK1*, *SP4*) displayed more than three instances across different BD‐cancer patients. Likewise, 11 genes (*SNCAIP*, *RAPGEF6*, *LARP1*, *FAXDC2*, *CNOT8*, *GEMIN5*, *MRPL22*, *KIF4B*, *SGCD*, *NEURL1B*) demonstrated recurring CNV duplications. These findings possess intriguing functional implications. For example, *KCND2* has been linked to the regulation of ERK signaling in ganglioglioma, with recurrent copy number breakpoints within *KCND2* associated with MET amplification [15]. Structural variants affecting *SNCAIP* disrupt the local chromatin environment, promoting abnormal gene induction, particularly PRDM6 in medulloblastoma [16]. Moreover, genes influenced by CNVs were identified based on experimental databases, and they were found to be associated with diseases and governed by impacted genes (Table 2).

**Table 2:** Genes with CNVs in BD‐cancer patients that identified with known regulated targets.

Gene ID	CNVs	Regulated targets	Targets associated diseases
IL6	Fully deletion	AU021063	Breast cancer
TLR3	Fully duplication	lnc‐IL7R	Oral squamous cell carcinoma
ELL2	Fully duplication	MAPK14	Malignant glioma
TSLP	Fully duplication	HOTAIR	Atherosclerosis
IRF1	Fully duplication	IRF1‐AS	Esophageal squamous cell carcinoma
EGR1	Fully duplication	FOXD2‐AS1	Gastric cancer, diabetes mellitus, lung adenocarcinoma, hepatocellular carcinoma
HDAC3	Fully duplication	LOC101928316	Gastric cancer
G3BP1	Fully duplication	H19	Hepatocellular carcinoma
BIRC7	Fully duplication	LNCRNA‐ATB	Kidney disease
REST	Partially duplication	ITIH4‐AS1	Colorectal cancer
PBX3	Partially duplication	SNHG10	Gastric cancer
KDM4C	Partially duplication	MALAT1	Colorectal cancer

Moving beyond individual genes, the study extended its focus to a functional network level (Fig. 4), addressing two pivotal questions: whether genes influenced by CNVs could cluster within the same biological pathways, and whether specific types of pediatric cancers exhibited overrepresentation in these pathway clusters. Compared to genes repeatedly affected by CNVs, functional pathways displayed higher prevalence among BD‐cancer patients (Table 3), suggesting robust heterogeneity at the gene level but more consistent patterns at the functional level. Numerous well‐known pathways related to tumor development and progression were identified, such as synapse organization (in 26 BD‐cancer patients, 14.4%), cadherin signaling (in 10 BD‐cancer patients, 5.5%), and the Wnt signaling pathway (in 13 BD‐cancer patients, 7.2%). This is significant, as recent studies have underscored the potential importance of synapse‐like structures in tumor cells and the tumor microenvironment—a phenomenon that could play a pivotal role in cancer cell metastasis [17].

**Table 3:** Number/percentage of BD‐cancer patients clustered based on functional pathways.

CNV types	Functional terms	Number of BD‐cancer patients	% in BD‐cancer patients have CNVs
All CNVs	GO0004984: Olfactory receptor activity	4	2.2
	GO0050808: Synapse organization	26	14.4
	GO0070851: Growth factor receptor binding	8	4.4
	GO0098742: Cell–cell adhesion	9	5.0
	KEGG: Basal cell carcinoma	8	4.4
	Kinase target: Casein kinase 1 delta	7	3.9
	PANTHER: Cadherin signaling pathway	10	5.5
	PANTHER: Wnt signaling pathway	13	7.2
	WikiCancer: Cytokines and inflammatory response	4	2.2
	WikiCancer: Endometrial cancer	5	2.8
	WikiCancer: Tumor suppressor activity of SMARCB1	6	3.3
Deletions	Disease: Autistic disorder	11	11.2
	Disease: Nerve sheath neoplasms	4	4.1
	GO0008238: Exopeptidase activity	2	2.0
	GO0016614: Oxidoreductase activity	4	4.1
	Locus: chr11q14	2	2.0
	Locus: chr22q13	3	3.1
	Locus: chr7p15	2	2.0
	Locus: chr7q32	1	1.0
	Locus: chr7q33	1	1.0
	miRNA target: MIR‐219	12	12.2
	miRNA target: MIR‐511	8	8.2
	WikiCancer: Tumor suppressor activity of SMARCB1	3	3.1
Duplications	Disease: Asthma	3	2.9
	Disease: Bronchial hyperreactivity	3	2.9
	GO0004984: Olfactory receptor activity	2	1.9
	GO0007606: Sensory perception of chemical stimulus	9	8.6
	GO0050808: Synapse organization	17	16.2
	GO0070851: Growth factor receptor binding	4	3.8
	GO0098742: Cell–cell adhesion	4	3.8
	KEGG: Olfactory transduction	4	3.8
	Kinase target: G protein‐coupled receptor kinase 3	2	1.9
	Locus: chr1q42	1	1.0
	Locus: chr1q44	3	2.9
	Locus: chr5q15	2	1.9
	Locus: chr5q23	3	2.9
	Locus: chr5q31	3	2.9
	Locus: chr5q32	2	1.9
	Locus: chr5q33	2	1.9
	miRNA target: MIR‐181	17	16.2
	miRNA target: MIR‐183	6	5.7
	miRNA target: MIR‐330	14	13.3
	PANTHER: Cadherin signaling pathway	7	6.7
	PANTHER: Wnt signaling pathway	7	6.7
	WikiCancer: Cytokines and inflammatory response	2	1.9

**Fig. 5:**
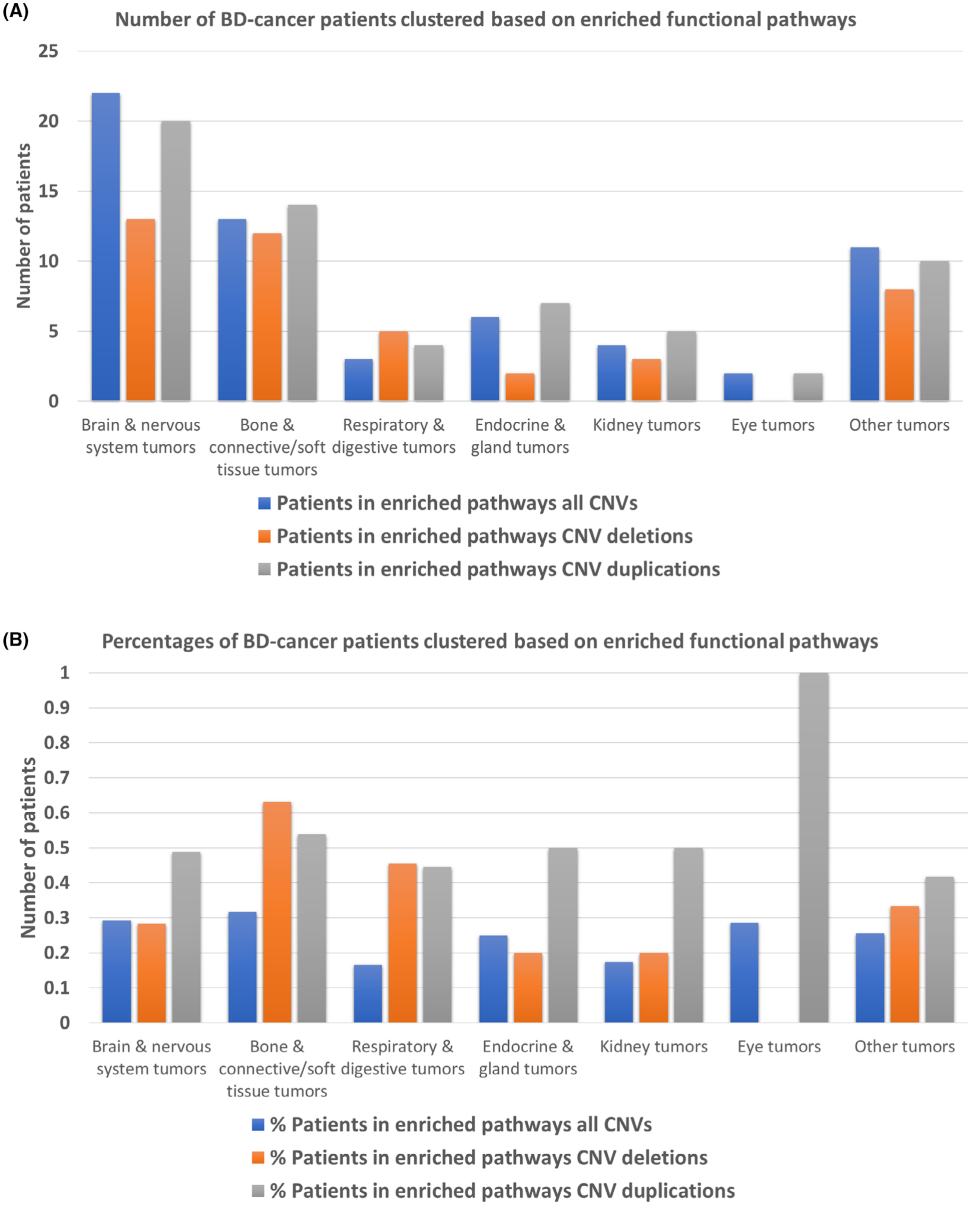
Birth defect (BD)‐cancer patients with subtype diagnosis clustered based on copy number variation (CNV) corresponding functional terms. (A) Number of patients in seven cancer subtypes clustered based on CNV corresponding functional terms; (B) percentage of patients in seven cancer subtypes clustered based on CNV corresponding functional terms.

Analyzing pediatric cancer subtypes, the study categorized them into seven groups: brain and nervous system tumors, bone and connective/soft tissue tumors, respiratory and digestive tumors, endocrine and gland tumors, kidney tumors, eye tumors, and other types. The analysis revealed distinct recurrence patterns within these cancer types, particularly in functional terms (Fig. 5). BD‐cancer children diagnosed with brain and nervous system tumors, as well as bone and connective/soft tissue tumors, displayed the highest ratios of pathway correspondences for CNVs—approximately 30%. Notably, nearly half of BD‐cancer patients exhibited duplications in enriched functional pathways, a common trait in tumors resulting from DNA damage in severe cancers. Remarkably, patients with malignant bone and connective/soft tissue tumors exhibited the highest ratios of CNV‐corresponding enriched pathways among all pediatric cancer types—both in deletions (63%) and duplications (54%). Given that CNVs have been identified as driver mutations in adult sarcomas and have prognostic and predictive potential for therapeutic response [5, 18], the deleted or duplicated genes in bone and connective/soft tissue tumors hold promise as blood biomarkers, meriting further exploration in pediatric sarcoma development.

Non‐coding RNAs have already demonstrated their critical roles in molecular functions and pathological implications in pediatric cancers [19, 20]. This study further established that CNVs within non‐coding RNAs exert significant impacts on tumor development processes in children with BD. The study identified a total of 932 long non‐coding RNAs, encompassing 262 CNV deletions across 82 BD‐cancer patients and 670 CNV duplications in 86 patients—constituting 33.7% of all BD patients. While only a small fraction (approximately 4%) of these long non‐coding RNAs could be mapped to their regulated targets based on functional research data, these 35 regulated targets consistently exhibited relevance to cancer (Table 1). Moreover, there was a significant enrichment of functional pathways associated with tumor development and progression (Fig. 3C,D). Among these, 35 lncRNAs with CNVs were identified in 12 patients, primarily within BD‐cancer patients diagnosed with brain and nervous system tumors, as well as bone and connective/soft tissue tumors (Fig. 4D). Notably, micro‐RNAs (MIRs) with CNVs were also identified as regulated targets in multiple BD‐cancer patients, demonstrating statistical significance (Table 3). This included MIR‐219, MIR‐511 (with deletions), and MIR‐181, MIR‐183, MIR‐330 (with duplications). MIR‐219, for instance, has been correlated with the prognosis of pediatric medulloblastoma [21], while MIR‐511 has demonstrated the ability to inhibit proliferation and invasion of osteosarcoma cells [22]. Similarly, MIR‐181 has been implicated in various cancers, and its presence has been linked to poor prognosis in neuroblastoma [23]. MIR‐330, acting as a tumor suppressor, regulates pediatric glioma cell proliferation and migration [24].

## 5 Conclusions

In conclusion, this study offers a comprehensive investigation into the impacts of CNVs within pediatric cancers in children with BD. The CNV detection described in this study is based on easily collectible blood samples. The identified CNVs corrected with pediatric cancers could serve as biomarkers for risk prediction of malignancies in children diagnosed with BDs. This could lead to increased attention for high‐risk children and enable early cancer interventions to prevent life‐threatening events.

## Conflict of interest

The authors declare no conflict of interest.

## Author contributions

YL, JG, and HH contributed to conceptualization; YL and JG contributed to literature search; YL, JG, H‐QQ, XC, FDM, TW, and HQ contributed to data preparation and analysis; YL, JG, H‐QQ, XC, TW, and HH contributed to data interpretation; YL contributed to original draft writing; and YL, JG, H‐QQ, and HH contributed to review and revision. All aspects of the study were supervised by HH.

### Peer review

The peer review history for this article is available at https://www.webofscience.com/api/gateway/wos/peer‐review/10.1002/1878‐0261.13718.

## Supporting information


**Table S1.** (a) ICD‐9/10 categories of birth defects; (b) ICD‐9/10 categories of birth defects with cancers.

**Table S2.** Copy number variations (CNVs) for individuals in this study.

**Table S3.** Gene lists for different types of copy number variations (CNVs).

**Table S4.** Long non‐coding RNA (lncRNA) lists for different types of copy number variations (CNVs).

## Data Availability

The KidFirst data could be accessed at Kids First Data Resource Portal (DRC) (https://portal.kidsfirstdrc.org/login).
